# The impact of shoe flexibility on gait, pressure and muscle activity of young children. A systematic review

**DOI:** 10.1186/s13047-019-0365-7

**Published:** 2019-11-29

**Authors:** Simone Cranage, Luke Perraton, Kelly-Ann Bowles, Cylie Williams

**Affiliations:** 1 Department of Physiotherapy, Monash University, Melbourne, Australia; 2 Department of Community Emergency Health and Paramedic Practice, Monash University, Melbourne, Australia; 3 Peninsula Health, Melbourne, Victoria Australia

**Keywords:** Shoes, Footwear, Gait, Child, Toddler, Walk, Run

## Abstract

**Background:**

There is limited evidence of shoe impact in younger children, particularly in the context of immature gait patterns. It is unclear if the impact from shoes in younger children is similar to what has been seen in older children. This systematic review aims to identify any impact of shoe features on younger children’s gait, and if there are any differences between shoe sole flexibility compared to barefoot.

**Methods:**

Study inclusion criteria included: typically developing children aged ≤6 years; comparison of barefoot and shod conditions (walking and/or running) with shoe features or style of shoe described; sample size > 1. Novelty types of footwear were excluded, as was any mention of in shoe support or modifications. Studies were located from six databases. Study methodology was assessed using the McMasters critical review form. Sample size weighted standardized mean differences (SMD) and 95% confidence intervals (CI) were calculated.

**Results:**

Four studies were included. Participant age ranged from 15.2 to 78.7 months, with 262 participants across all studies. All studies had limited methodological bias based on their design type. Compared to barefoot walking, shoes increased velocity, step time and step length. Shod walking decreased cadence. Peak plantar pressure was generally lower in the stiff shoe design and there was a higher peak plantar pressure in the Ultraflex shoes. No studies were found investigating muscle activation.

**Conclusions:**

Shoes affect younger children’s gait in spatiotemporal gait aspects, similar to those seen in older children. There is limited evidence on effects of particular shoe features such as sole hardness, on gait, and no evidence of any changes in muscle activation patterns. Further research is required to evaluate the impact of different types of shoe and shoe features in this population to provide clinical advice on the type of shoe that is appropriate in this age group.

## Background

Mature gait patterns are well established in children by the age of 3 years [1]. Typical indicators of the establishment of mature gait include the presence of a reciprocal arm swing and heel strike. There is also an increase in velocity, step length and single support together with a reduction in cadence [1]. Studies observing the gait of children between the ages of 1 to 10 years have found that normalised velocity and step length increases gradually from 1 to 4 years and stabilises between 5 to 10 years of age [2]. Young children’s walking and running is often less stable and less efficient than that of older children and adults due to a higher centre of gravity; lower muscle to body weight ratio; an immature nervous system and poorer postural control [1].

Health professionals and members of the public often advise parents to allow their toddlers to be barefoot as much as possible, or to wear soft soled shoes in the early developmental stages of walking [3]. This is thought to allow an increase in muscle strength in their feet and to assist in sensory experiences with different surfaces. Health professionals and shoe manufacturers often give advice based on the assumption that a shoe should not affect normal foot function or motor development in younger children and therefore be as close to barefoot walking as possible [3]. However, there is limited research evidence to guide these shoe recommendations in younger children.

There is also limited research to guide health professionals on the impact of shoes on the gait of children. This is predominantly in children over the ages of six years [4]. Older children walking in shoes resulted in an increased walking velocity, longer stride length, increased stride time, decreased cadence, wider base of support, later toe off time during the gait cycle, increased double support time and a longer stance time, than when walking barefoot [5].

There has also been an observation of changes in lower limb kinetics with shoes changing tibialis anterior activity (compared to barefoot in children with a mean age of 7.7 years (range 2–15 years) [6]. In another study, shoes were also noted to decrease the intrinsic motion of the foot, which could indicate possible splinting effect of shoes on foot joints, a study undertaken with children aged above six years [7].

There is limited available evidence on shoe impacts in younger children, particularly in the context of an immature gait pattern. It is particularly unclear if there are similar impacts from shoes in younger children as seen in their older counterparts. The primary aim of this systematic review was to examine the impact of shoe features on younger children’s gait. The secondary aim was to investigate any differences between shoe sole flexibility compared to barefoot gait.

## Method

### Search strategy

This review was undertaken in accordance with the Preferred Reporting Items for Systematic Reviews and Meta-Analysis (PRISMA) guidelines [8]. Two reviewers (SC, CW) examined six databases from inception to April 2018. Databases searched were: OVID Medline, EMBASE, CINAHL, EBM reviews, AMED and Sports Discus. Search terms included synonyms of: child, infant, pediatric, gait, walk, jog, run, ambulation stride, step, swing, pressure, force, kinematics, kinetics, angle, spatiotemporal, EMG, electromyography, gait, GAITrite, Trigno, footwear, shoe$, trainer$, sole, boot$, sandal$, stiffness, hardness, Velcro, buckle, lace, fasten* (Limiter for full text publications and human studies). Boolean operators “AND” and “OR” were used to combine search terms relating to the search question. Where search term variations existed, truncation (*) was used. All research designs were included. An example search strategy for Ovid Medline is outlined (Table 1). Studies were only included if they were published in a peer reviewed journal.

**Table 1:** Search and selection process for the review studies

1.	Child
2.	Infant
3.	P(a)ediatric
4.	Walk
5.	Jog
6.	Run
7.	Ambulat$
8.	Stride
9.	Step
10.	Swing
11.	Pressure
12.	Force
13.	Kinematic$
14.	Spatiotemporal
15.	Electromyography
16	Gait
17.	Trigno
18.	Footwear
19.	Trainer$
20.	Sole
21.	Boot$
22.	Sandal$
23.	Stiff*
24.	Hard*
25.	Velcro
26.	Buckle
27.	Lace
28.	Fasten*
29.	1 or 2 or 3
30.	4 or 5 or 6 or 7 or 8 or 9 or 10 or 11 or 12 or 13 or 14 or 15 or 16 or 17
31.	18 or 19 or 20 or 21 or 22 or 23 or 24 or 25 or 26 or 27 or 28
32.	29 and 30 and 31
33.	Limit 32 to human

### Eligibility criteria and screening

Prior to searching, the research team determined inclusion and exclusion criteria for the study (Table 2). Duplicates were removed from the search yield via Endnote and two authors (SC and CW) independently screened the abstracts of all retrieved studies against the eligibility criteria using Covidence [9]. Articles were included for full text review where there was uncertainty from the abstract.

**Table 2:** Eligibility criteria

Inclusion criteria	Exclusion criteria
Children aged ≤6 years	Articles with full text not published in English
Comparison of barefoot and shod conditions (walking and/or running)	Novelty types of footwear
Typically developing children	Orthoses, arch supports or innersoles mentioned
No identified pathology known to impact on gait Sample size of total participants > 1	Children having a medical condition known to impact on gait
Shoe features or style described	

Two authors reviewed the title and abstract (SC, CW) to determine if the study was to be included in a full text screening. Any differing opinions were discussed and resolved in person. In cases of non-consensus, a third author’s opinion was planned for consultation; however, this was not required. All citations of included articles and reference lists were also screened against the eligibility criteria and any articles meeting the inclusion criteria were also included within this review.

### Risk of bias assessment-

All articles included within the final review underwent methodological assessment using the McMaster critical review form- Quantitative studies [10] which is applicable to Randomised Controlled Trials, controlled trials and cross sectional intervention trials. The tool has fifteen individual assessment points within eight domains. Risk of bias was completed independently by two reviewers (SC and LP) and achieved consensus with further discussions and review from a third and fourth reviewer where required (CW, KB).

### Data management

Where data suitable for extraction was not available, authors were contacted to provide unpublished data. If there was no response within 4 weeks, these articles were excluded from the final review. Data describing the study sample characteristics; study design; shoe design and features; spatiotemporal measures; and kinetics were extracted by two reviewers independently. Consensus on results was discussed between two reviewers who extracted the data (LP and SC). Means and standard deviations for each group were extracted where data was provided or supplied on request.

### Statistical methods

Participant characteristics were described by means, standard deviations (SD) and frequencies (%). Data were extracted from each study by age, and where there was greater than one participant per age and per condition gait variables were included for meta-analysis. To satisfy the assumption of independence only the data from right side were used within meta-analysis [11]. Where only means and confidence intervals were reported, the group standard deviations were calculated as per the formula SD = √N x (upper limit – lower limit)/3.92. Sample size weighted standardized mean differences (SMD) and 95% confidence intervals (CI) for gait variables were calculated using Stata 13 (StataCorp LP.) with the differences in mean scores between the shoe groups and the mean standard deviation using a random effect model (Mantel-Haenszel method) to account for the use of paired data. SMDs were considered to be statistically significant if their associated CI did not cross zero. Interpretations of strength of the SMDs statistics were based on Cohen’s guidelines with small effect ≥0.2, medium effect ≥0.5, and large effect ≥0.8 [12].

## Results

### Study selection and design

A total of 4037 articles were screened by two independent reviewers (SC, CW). Thirty two studies were included for full text screening based on the eligibility criteria. Five studies met the inclusion criteria and were included in the final review. The search and selection process of the articles is described in Table 1.

One study was subsequently excluded, as the data were only aggregate data reported for children between five to 11 years [13]. Gait variables for the five and six year old children within this paper were unable to be separated from the data of children aged seven and above. The author was contacted however no response was received.

#### Characteristics of included studies

Table 3 includes the characteristics of the four included papers. All were cross sectional studies (Level IV evidence on the NHMRC evidence hierarchy). The age of the participants in the included studies ranged from 15.2 months to 78.7 months and there were a total 262 participants in the four studies. Table 3 also provides the gait variables per age and per condition.

**Table 3:** Description and methodological approach of studies included in review

Author	Country	Design	Sample size	Gender (Female) n (%)	Mean age (SD)months	Gait type	Shoe conditions included in analysis	Outcome measures
Buckland, 2014	USA	Cross sectional Repeated measures	25	8 (32%)	15.2 (2.0)	Walk	Lace up sneakers (Ultraflex, Medflex, Lowflex, Stiff)	Spatiotemporal
Hillstrom, 2013	USA	Cross sectional Repeated measures	24	8 (32%)	15.2 (2.0)	Walk	Lace up sneakers (Ultraflex, Medflex, Lowflex, Stiff)	Plantar pressures
Lythgo, 2009	Australia	Cross sectional	69 (5 years)	33 (48%)	68.4 (0.2)	Walk	Athletic shoes/runners (own)	Spatiotemporal
		Repeated measures	140 (6 years)	75 (54%)	78.7 (0.3)	Walk		
Kennedy, 2018	Australia	Cross sectional	1 (4 years)	0 (0%)	50.9	Walk	Optimal (runners) own shoes/sub optimal (flip flops) own shoes	Spatiotemporal
		Repeated measures	3 (5 years)	2 (67%)	62.8 (5.16)			

There were three studies examining the spatiotemporal features of gait [5, 14, 15] and one that investigated pressure [16]. There were no studies found that investigated muscle activity. All four included studies examined gait while the young children wore athletic type shoes and compared gait in these to barefoot. Two studies, with the same cohort of participants, standardized the torsional flexibility of the shoes [14, 16]. The torsional flexibility was assessed by determining the amount of force required to cause angular rotation on each shoe, and results classified shoes into; Ultraflex, Medflex, Lowflex and stiff [14, 16]. One of these two studies evaluated spatiotemporal measures during walking [14], while one study evaluated plantar pressures during walking [16] and reported the data on the same cohort of children. Running was not assessed in any of the included studies.

### Spatiotemporal findings

**Fig. 1:**
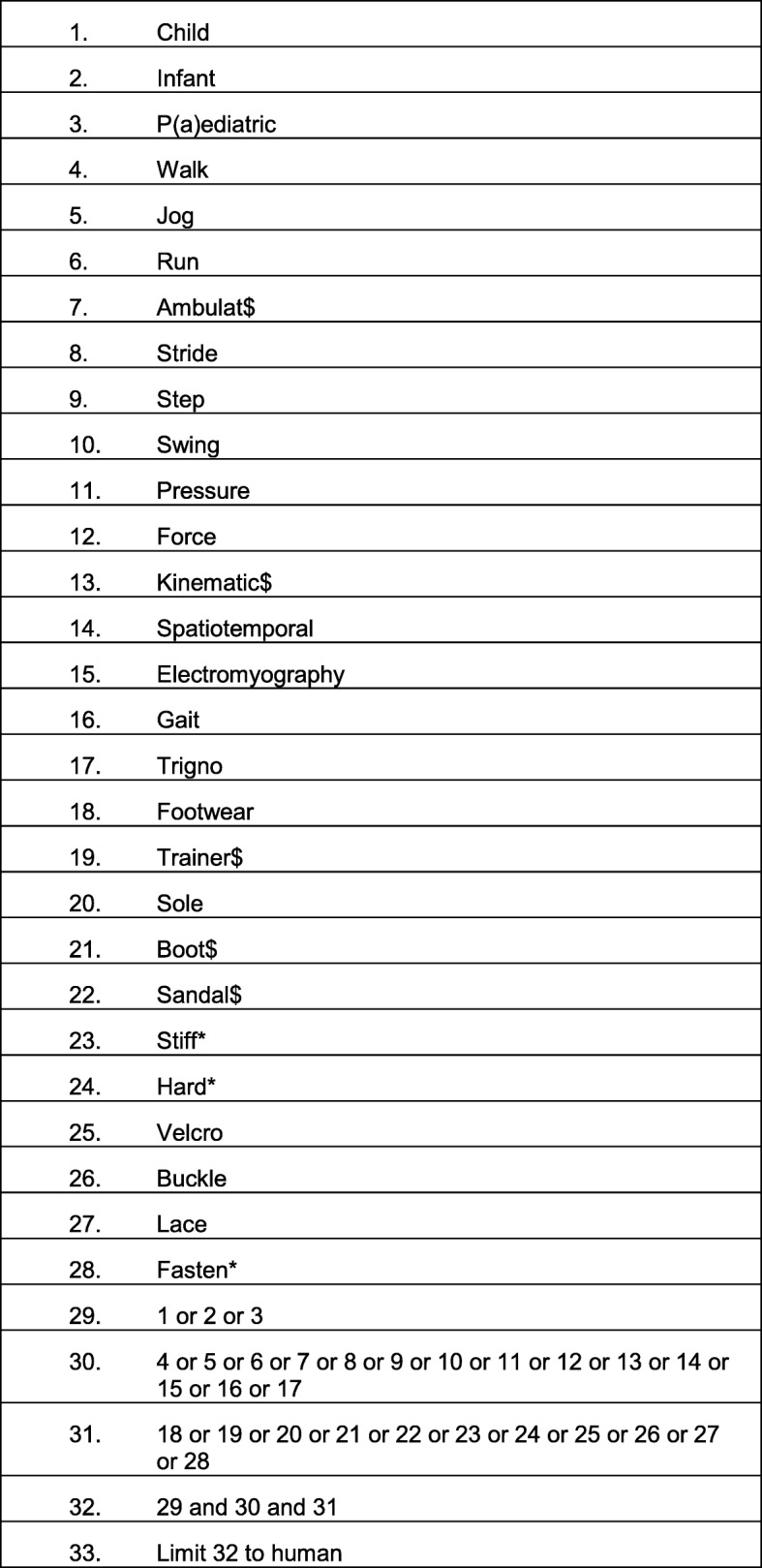
Forest plots of the differences in **a**) velocity, **b**) cadence, **c**) step length **d**) step time differences between shoes compared to barefoot walking for young children

There were three studies that reported spatiotemporal changes for barefoot versus shoes (Table 4). Two studies had data available for similar ages and were used within a meta-analysis for the variables velocity, cadence, step time and step length (Figs. 1a, b, c and d). Compared to barefoot walking, shoes decreased cadence (SMD = − 2.50, 95%CI = -3.45,-1.54, I^2^ = 87.2%), increased step time (SMD 1.44, 95%CI = -0.04, 2.91, I^2^ = 95.8%increased step length (SMD = 5.60, 95%CI = 4.66, 6.55, I^2^ = 66.4%) and may increase velocity, (SMD = 1.65, 95%CI = 0.74, 2.56, I^2^ = 89.9%),

**Table 4:** Velocity, cadence, step time and step length data included within meta-analysi

Author	Mean (SD) Age month	Conditions	Velocity mean (SD), cm/sec)	Cadence mean (SD), steps/min	Step Time (cm/sec) Right only	Step length (cm) Right only
Buckland, 2014	15.2 (2.0)	Barefoot	87.3 (19.7)			26.30 (4.30)
Buckland, 2014	15.2 (2.0)	Lace up sneakers (Ultraflex)	87.70 (18.90)			28.10 (3.70)
Buckland, 2014	15.2 (2.0)	Lace up sneakers (Medflex)	85.70 (19.10)			28.10 (5.70)
Buckland, 2014	15.2 (2.0)	Lace up sneakers (Lowflex)	83.10 (19.30)			27.40 (4.40)
Buckland, 2014	15.2 (2.0)	Lace up sneakers (Stiff)	86.00 (16.20)			28.10 (3.70)
Lythgo, 2009	68.4 (0.2)	Barefoot	124.80 (4.60)	152.60 (4.10)	389 (11)	48.70 (1.10)
Lythgo, 2009	78.7 (0.3)	Barefoot	127.5 (2.40)	146.30 (2.60)	415 (8)	52.20 (0.90)
Lythgo, 2009	68.4 (0.2)	Athletic shoes/runners (own)	130.30 (4.20)	142.80 (3.40)	423 (10)	54.80 (1.20)
Lythgo, 2009	78.7 (0.3)	Athletic shoes/runners (own)	133.50 (2.80)	138.40 (2.10)	437 (10)	57.80 (0.90)
Kennedy, 2018	50.9	Barefoot	114.8	157.1	378	44.23
Kennedy, 2018	66.8 (5.1)	Barefoot	113.7 (14.3)	147.5 (15.8)	410 (45.1)	46.38 (1.3)
Kennedy, 2018	50.9	Optimal (runners)	139.9	158.3	377	53.23
Kennedy, 2018	66.8 (5.1)	Optimal (runners)	126.0 (9.6)	140.4 (14.6)	430 (42.7)	54.42 (2.8)

### Plantar pressures

Peak plantar pressures were significantly different across four differing shoe conditions relating to stiffness of shoe sole [16]. Overall, the peak plantar pressure was generally lowest in the stiff shoe design (mean = 9.6, SD = 3.2 N/cm2) and there was a higher peak plantar pressure in the Ultraflex shoes (mean = 13.0, SD = 3.8 N/cm2).This was the only study that examined pressure variables [16].

### Study quality (risk of bias assessment)

A quality assessment of the articles was completed to assess the risk of bias with the McMaster quantitative critical appraisal tool [10]. All domains were scored for each of the included articles (Table 5). Three studies did not provide a justification of their sample size [14–16]. The clinical importance and clinically meaningful difference between groups were unable to be concluded due to low power within one study [14]. All of the studies included within the review showed good methodology quality for their design type.

**Table 5:** Methodological quality of the studies included in the review as assessed by the McMasters Quality Assessment

Study	1	2	3	4	5	6	7	8	9	10	11	12	13	14	15	Score
Buckland [14]	1	1	1	0	1	1	1	1	1	1	1	0	0	1	1	12/15
Hillstrom [16]	1	1	1	0	1	1	1	1	1	1	1	0	0	1	1	12/15
Lythgo [5]	1	1	1	1	1	1	1	1	1	1	1	1	1	1	1	15/15
Kennedy [15]	1	1	1	0	1	1	1	1	1	1	1	1	1	1	1	14/15

## Discussion

The findings of this systematic review are the first to examine the impact of shoes on young children. The previous systematic review of children between the ages of 1.6 years and 15 years, found that children wearing shoes, walk faster by taking longer steps, with an increase in the support phases of the gait cycle [4]. These gait changes may result from footwear increasing the leg length related to the shoe, or an increased mass of the shoe increasing inertia of the leg during the swing phase [4]. The gait changes observed in older children were similar to those observed in younger children within this systematic review. Given the lightweight nature and low shoe base to upper ratio of young children’s shoes, it is unknown if shoe height and mass also contribute to these changes in younger children’s gait.

The variability of shoe advice from health professionals may be challenging for parents [17, 18]. Previous studies have described optimum foot development occurring in a barefoot environment with the primary role of shoes being to protect the foot from injury [3]. The results from this systematic review indicate there is an absence of evidence to support one shoe type over another, and limited evidence that shoe flexibility has an impact on young children’s gait. While shoes appear to have some influence on gait parameters, it is not yet known if these changes effect function or have any long-term effects on foot health. There are also consistent messages to parents that a stiff and compressive shoe may cause deformity, weakness and loss of mobility [3]. In spite of these negative messages, there are no consistent international and evidence-based recommendations to guide clinicians or manufacturers on the optimal shoe for younger children, in particular whether a child should wear a soft or hard-soled shoe. It is unfortunate that the results of this review indicate that more research is needed rather than providing credible evidence to support either of these recommendations.

This absence of evidence supporting shoe recommendations for children is also a challenge when clinicians are presented with children who have a pathological gait or a foot or lower limb concern. If there is limited literature on typically developing children and shoes, it is difficult to compare the impact of shoes on children with pathological gait. The findings of this review will hopefully encourage future research into the effects of shoe sole features on the gait parameters in children; therefore helping to guide clinicians and shoe industries on the appropriate shoe for younger children.

There are a number of limitations within this review including the limited number of available studies for analysis. It is unknown if the lack of studies is correlated to the challenges that present while testing this age group of children. The psychosocial challenges of having children within a gait laboratory environment is proposed as a large contributing factor to the limited number of studies available to base this review on.

Often gait analysis methods rely on placing markers on small children, which potentially can cause the child to subtly change gait, particularly in young children. The gait environment is also an unappealing play environment therefore challenging to provide ongoing motivation for a younger child to complete all tasks in order to obtain a complete data set during testing.

There was also a limitation in the variability of the shoe and limited descriptions. Like adults, young children wear a variety of shoes including athletic shoes, sandals or boots. It is unknown if the variation in shoe type and their features also contribute to the differences in gait. An additional limitation is the limited number of studies included within the meta-analysis. One of the included studies had a small sample size of four participants for the age range we were interested in [15], therefore, caution should be applied to these results. All full text articles were limited to English which is another limitation of this study.

Further research is required for health professionals to provide recommendations on the optimal shoe characteristics for younger children, including sole hardness. Prospective research is required to determine whether shoe and sole flexibility lead to changes in kinetics, kinematics and muscle activation patterns in younger children and whether changes associated with shoes are associated with clinical and patient-reported outcomes.

## Conclusion

Shoes affect the gait of young children by increasing velocity, cadence, step time and step length compared to bare feet, similar to that of older children. There is limited evidence on the effect of particular shoe features such as sole hardness, on gait and no evidence on any changes in muscle activation patterns. Further research is required to evaluate the impact of different types of shoe and shoe features in this population to provide clinical advice on the type of shoe that is appropriate in this age group.

## Data Availability

Please contact author for data requests.
