# Correction to: The impact of shoe flexibility on gait, pressure and muscle activity of young children. A systematic review

**DOI:** 10.1186/s13047-019-0368-4

**Published:** 2020-01-20

**Authors:** Simone Cranage, Luke Perraton, Kelly-Ann Bowles, Cylie Williams

**Affiliations:** 1 Department of Physiotherapy, Monash University, Melbourne, Australia; 2 Department of Community Emergency Health and Paramedic Practice, Monash University, Melbourne, Australia; 3 Peninsula Health, Melbourne, Victoria Australia

**Correction to: J Foot Ankle Res (2019) 12:55**

**https://doi.org/10.1186/s13047-019-0365-7**

After publication of our article [1] we were notified that Fig. 1 was incorrectly published as a duplicate of Table 1. The updated Fig. 1 is included in this correction.

**Fig. 1:**
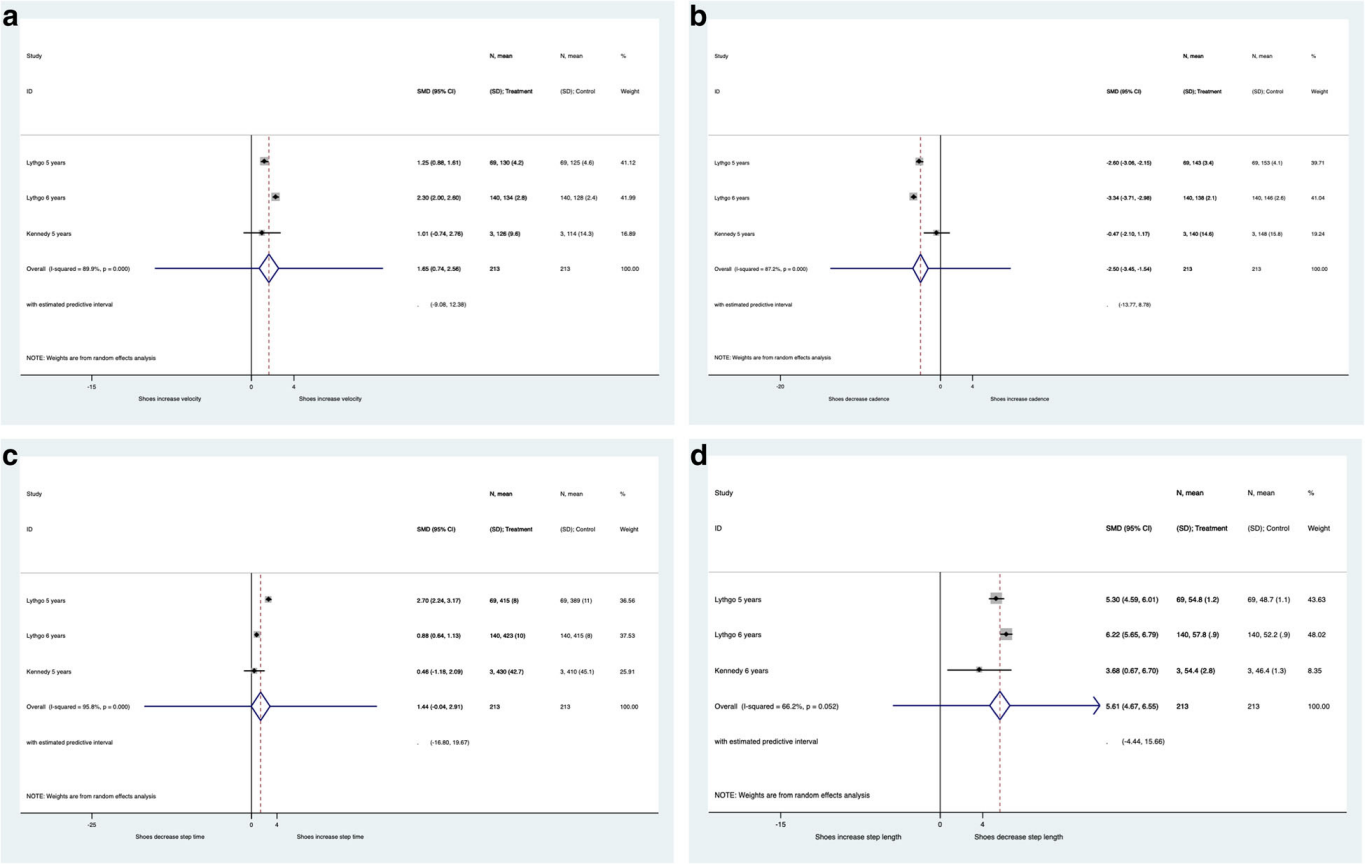
Forest plots of the differences in **a**) velocity, **b**) cadence, **c**) step time **d**) step length differences between shes compared to barefoot walking for young children

The original article has been corrected.
